# Ultra-high resolution magnetic resonance microscopy of *in situ* gadolinium gold nanoparticle-labeled cells in the rat brain†

**DOI:** 10.1039/d5sc01588j

**Published:** 2025-06-03

**Authors:** Alena Kisel, Minrui Luo, Matthew D. Bailey, Harman Ghuman, Matthew Rotz, Vinícius P. Campos, Marcelo A. C. Vieira, T. Kevin Hitchens, Thomas J. Meade, Michel Modo

**Affiliations:** a Department of Radiology, McGowan Institute for Regenerative Medicine, University of Pittsburgh 3025 East Carson St Pittsburgh Pennsylvania USA mmm154@pitt.edu +1 (412) 383 7200; b Department of Bioengineering, University of Pittsburgh Pittsburgh Pennsylvania USA; c Department of Neurobiology, University of Pittsburgh Pittsburgh Pennsylvania USA; d Department of Chemistry Evanston Illinois 60208 USA tmeade@northwestern.edu +1 (847) 491 2481; e Department of Neurobiology Evanston Illinois USA; f Department of Molecular Biosciences, Northwestern University Evanston Illinois USA; g Department of Electrical and Computer Engineering, São Carlos School of Engineering University of São Paulo São Carlos SP Brazil

## Abstract

Mapping the distribution of cells within a tissue using MR imaging has remained a significant challenge for the field. Cellular MRI can trace cells within tissue, but typically does not achieve the resolution necessary to define a cell's precise anatomical location. To detect cells with ultra-high resolution MRI, a high *r*_1_ relaxivity intracellular contrast agent is required. Localizing this contrast within its biological context also necessitates an isotropic spatial resolution corresponding to the size of a cell's cytoplasm (∼20 μm) to place it within its biological context. We here demonstrate that gadolinium gold nanoparticles (GdAuNP) induce a high T_1_-weighted cellular MRI contrast at ultra-high magnetic fields (9.4 T, and 11.7 T) that affords *in situ* labelled cell detection at very high resolutions (150, 100, 50, and 20 μm). A 20 μm 3D gradient-echo image (400 minutes scan) combined with MR image denoising robustly visualized the distribution of *in situ* labeled cells in the rat brain. Signal averaging (NA = 5) also consistently afforded the detection of labeled cells. Positive T_1_-weighted contrast was confirmed to be caused by GdAuNP using histology. Immunohistochemistry confirmed the presence of GdAuNP almost entirely inside cells, primarily those of the neuronal lineage. Histology verified that the MR images accurately visualized individual cells' distribution within their anatomical context. Cellular resolution MRI of GdAuNP-labeled cells hence affords new avenues to investigate how individual cells contribute to the development, repair, and regeneration of tissues.

## Abstract 2

Mapping the distribution of cells within a tissue using MR imaging has remained a significant challenge for the field.

## Graphical abstract

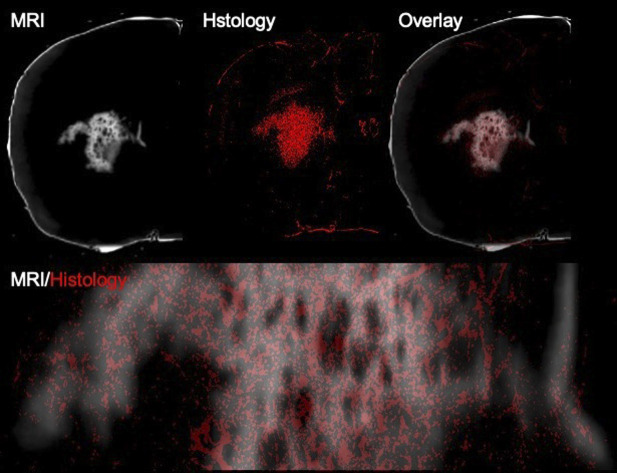

## Introduction

^1^H Magnetic Resonance Imaging (MRI) has excellent soft tissue contrast due to its high water density.^1^ Akin to histology, to visualize the distribution or migration of cells, however, an exogenous contrast material, such as iron oxide or gadolinium-based contrast agents, is required to distinguish the cells of interest from their surrounding tissue.^2^ Cellular MRI has been used to track the migration of stem cells, as well as distinguish different cellular fractions within the liver.^3^ The MR detection of single micron-sized particles of iron oxide (MPIOs) is feasible^4^ and affords the detection of single cells.^5,6^ The superparamagnetic effects of iron oxide cause local magnetic field gradients that are observed as areas of signal hypointensities on 
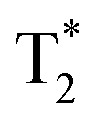
-weighted images. This does not only affect the immediate location of the contrast particle, but the so-called blooming effect can extend over 50× the particle size.^7,8^ The presence of multiple cells can, hence, lead to a total signal loss in tissue and prevent the anatomical localization of the cell(s) of interest. To localize a cell to an individual voxel, negative image contrast and blooming effects should, therefore be minimized.

Unequivocal positive contrast confined to a single voxel would be desirable to localize cells in their anatomical context. For instance, T_1_-weighted (T_1_w) MRI can accurately detect *in vivo* neural stem cells (NSCs) implanted in the rat brain using hyperintense contrast due to their *in vitro* labeling with gadolinium-gold nanoparticles (GdAuNP).^9^ Conjugation of gadolinium chelates to gold nanoparticles is an efficient means to dramatically increase and maintain their T_1_ relaxivity upon intracellular uptake, as well as to improve their thermokinetic stability in a biological milieu.^10–12^ Attachment of DNA on GdAuNP improves cellular uptake and traps nanoparticles in a peri-nuclear location, which prevents exocytosis and transfer to other cells.^9,13^ A highly selective T_1_w visualization of GdAuNP-labeled cells after intracerebral implantation can hence be achieved *in vivo* whilst affording their anatomical localization.^9^

Although the blooming effect of iron oxide particles has afforded the detection of single cells/particles by MRI at a 100 μm resolution.^5,6^ This cellular MRI to date has not achieved a cellular resolution that would afford a precise localization of a cell's positioning, especially amongst a population of contrast agent-labeled cells. To achieve a cellular resolution for a population of contrast agent-labeled cells, MRI will require a spatial resolution that can potentially detect a cell within one isotropic voxel. We previously measured *in vitro* the average diameter of NSCs to be 19.29 ± 0.75 μm.^14^ We here therefore propose that an isotropic voxel size ≤20 μm is required to achieve a cellular-resolution MRI, a resolution 125× smaller than previously used to detect individual cells labeled with iron oxide-based contrast agents.^6,15,16^ Isotropic resolutions <100 μm are considered ultra-high resolution and consequently are often referred to as MR microscopy (MRM).^17^

Achieving a cellular resolution MRM will be important to monitor the distribution and migration of a population of transplanted cells through a tissue,^18^ and also provide deeper insights into the spatio-temporal dynamics of neurogenesis and its response to tissue damage.^19–21^ Considering the widespread migration of NSCs, as well as the varied topology of brain damage, as occurs after a stroke or traumatic brain injury, non-invasive whole brain imaging is required to monitor the distribution of cells. We here demonstrate that GdAuNP labeling of cells *in situ* combined with a 20 μm isotropic resolution affords a cellular resolution MRM to investigate the distribution of individual cells in the neurogenic subventricular zone (SVZ), as well as within striatal tissue.

## Results

### Optimization of T_1_-weighted MRI to distinguish GdAuNP from brain tissue

GdAuNP are designed to achieve a high relaxivity that is efficiently delivered into cells for cellular MRI. The highly negative charge of ssDNA renders AuNPs exceptionally stable. The ssDNA facilitates endocytosis and the localization of GdAuNP in the peri-nuclear space within cells. The random DNA sequence cannot integrate into the nuclear DNA and hence traps the GdAuNP in the peri-nuclear space. By Gd-labeling ssDNA on AuNPs and labeling the remaining nanoparticle surface with additional Gd, we are able to achieve a GdAuNP *r*_1_ of over 3300 mM^−1^ s^−1^. These ssDNA-GdAuNP are suitable for long-term cellular imaging studies, as their subcellular localization and anchoring prevents their clearance from labeled cells and provides a signal specific to these cells. However, to efficiently exploit their excellent relaxivity and labeling, optimal MRI acquisition parameters are required.

**Fig. 1:**
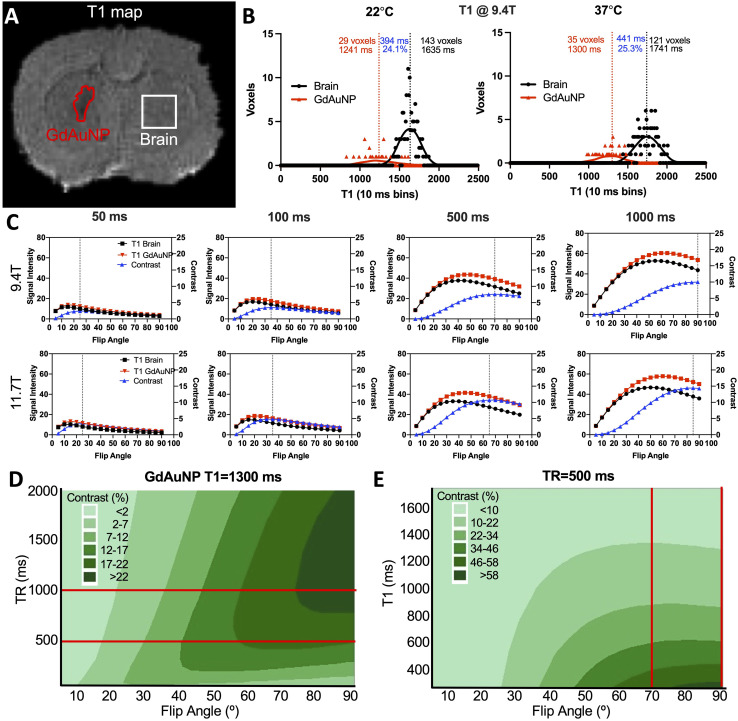
Optimization of T_1_ acquisition parameters. (A) To calculate the impact of GdAuNP on the T_1_ signal in brain tissue, regions-of-interest (ROIs) corresponding to brain tissue and the signal produced by GdAuNP-labeled neural stem cells were measured on a T_1_ map. (B) Scanning at both room temperature (22 °C) and physiological temperature (38 °C) afforded mapping of T_1_ for individual voxels of brain tissue and GdAuNP-labelled cells under two common experimental conditions (*i.e. ex vivo* and *in vivo*). T_1_ at 38 °C was marginally higher for both brain tissue (6.1%) and GdAuNP (4.6%). (C) Based on T_1_ measurements of brain tissue and GdAuNP (22 °C) at both 9.4 T and 11.7 T, optimal parameters for T_1_ acquisition were calculated using the Ernst equation for 4 different repetition times (TR, 50, 100, 500, 1000 ms). Optimal parameters were derived from maximum T_1_w contrast rather than peak signal intensity for brain tissue or GdAuNP. With shorter TR, the optimal flip angle (FA) was lower, whereas with a TR of 1000 ms optimal flip angle was 90°. A disadvantage of longer TR is longer acquisition times. (D) To map the experimental space of TR and FA, contour maps were computed to illustrate their interaction and provide a means to choose optimal TR and FA to detect maximum contrast between GdAuNP and brain tissue. A TR of 1000 ms with a FA > 75° achieves maximum contrast (>22%), whereas a shorter TR of 500 ms achieves a lower contrast (17–22%), while affording twice the number of signal averages. (E) To illustrate how a GdAuNP decrease in T_1_ affects contrast, a contour map of decreasing T_1_ demonstrates that with a FA of 70°, T_1_ contrast ranging from 10 to >46% can be achieved if sufficient contrast agent concentration is present.

Measurement of T_1_ relaxivity *in situ* of GdAuNP-labeled cells afforded a specific intracellular environment to optimize contrast between labeled cells and brain tissue (Fig. 1A). GdAuNP decreased T_1_ by 24.1% at room temperature (22 °C) and by 25.3% at physiological temperature (37 °C) (Fig. 1B). Based on these measurements, optimal flip angles (FA) were calculated for different repetition times (TR) using the Ernst equation at 9.4 T and 11.7 T (Fig. 1C). A contour plot arraying both FA and TR revealed an optimal TR = 1000 ms with a FA of 90° achieving a T_1_ contrast >22% (Fig. 1D). However, a shorter TR = 500 ms with an FA of 70° already achieved a contrast >17% within half the acquisition time, providing a faster acquisition and the opportunity for more signal averages within the same unit of time. A greater reduction in T_1_ due to GdAuNP relaxivity further translates into a greater T_1_ contrast between GdAuNP and brain tissue with these optimized acquisition parameters (Fig. 1E).

**Fig. 2:**
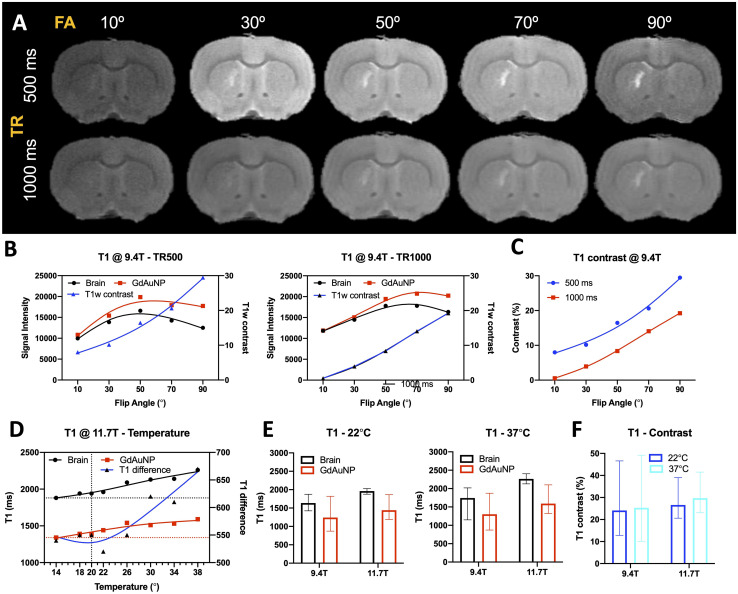
Impact of field strength and sample temperature on T_1_ acquisition. (A) Experimental verification of optimal acquisition parameters visually confirmed a robust detection of GdAuNP with a TR = 500 ms and FA > 70°, as well as TR = 1000 ms and FA = 90°. (B) Quantification of signal intensities for brain tissue and GdAuNP, as well as the contrast between both further illustrate how optimization improves T_1_w contrast. (C) A direct comparison between optimized parameters of a TR of 500 and 1000 ms indicated a consistently greater (6–10%) contrast with the TR = 500 ms. A maximum of 29.5% contrast was achieved with TR = 500 ms and a flip angle of 90°. (D) To systematically investigate the impact of sample temperature and the Curie Point of gadolinium on T_1_w contrast, sample temperature was controlled during scanning in a 11.7 T MRI scanner. T_1_ of both brain tissue and GdAuNP signals increased by 16% in a linear fashion between 14 and 38 °C. The T_1_ difference between both increased with rising temperatures >22 °C. Maximum T_1_ difference was achieved with a physiological temperature of 37 °C, indicating that even scanning *ex vivo* samples at physiological temperature being preferable to room temperature (22 °C). (E) T_1_ measurements of GdAuNP were increased by 14% (22 °C) and 18% (38 °C) at 11.7 T compared to 9.4 T. (F) However, T_1_ contrast (*i.e.* the difference between brain tissue and GdAuNP) was only improved by 4% between 9.4 T and 11.7 T.

An experimental verification of these parameters was achieved by injection of GdAuNP into two distinct microenvironments, notably the lateral ventricle of a rat brain (liquid environment with abundant access to water), as well as rat striatal tissue (limited water access and increased cellular uptake). Strong contrast was evident at both TR = 500 ms with FA > 70°, as well as TR = 1000 ms with an FA of 90° (Fig. 2A). This was further confirmed by quantification of contrast revealing up to 30% T_1_w contrast with a TR = 500 ms and 20% with a TR = 1000 ms (Fig. 2B). The T_1_w contrast for TR = 500 ms was consistently ∼10% higher than for TR = 1000 ms (Fig. 2C). The T_1_ signal of both GdAuNP and brain tissue was increased with rising temperature, achieving a higher signal difference at physiological temperature (37 °C) (Fig. 2D). Field strength further amplified the effects of temperature on T_1_ signal measurements (Fig. 2E). However, T_1_w contrast between GdAuNP and brain tissue was consistent at 22 °C and 37 °C, as well as at 9.4 T and 11.7 T (Fig. 2F).

### Achieving a cellular resolution MR microscopy

**Fig. 3:**
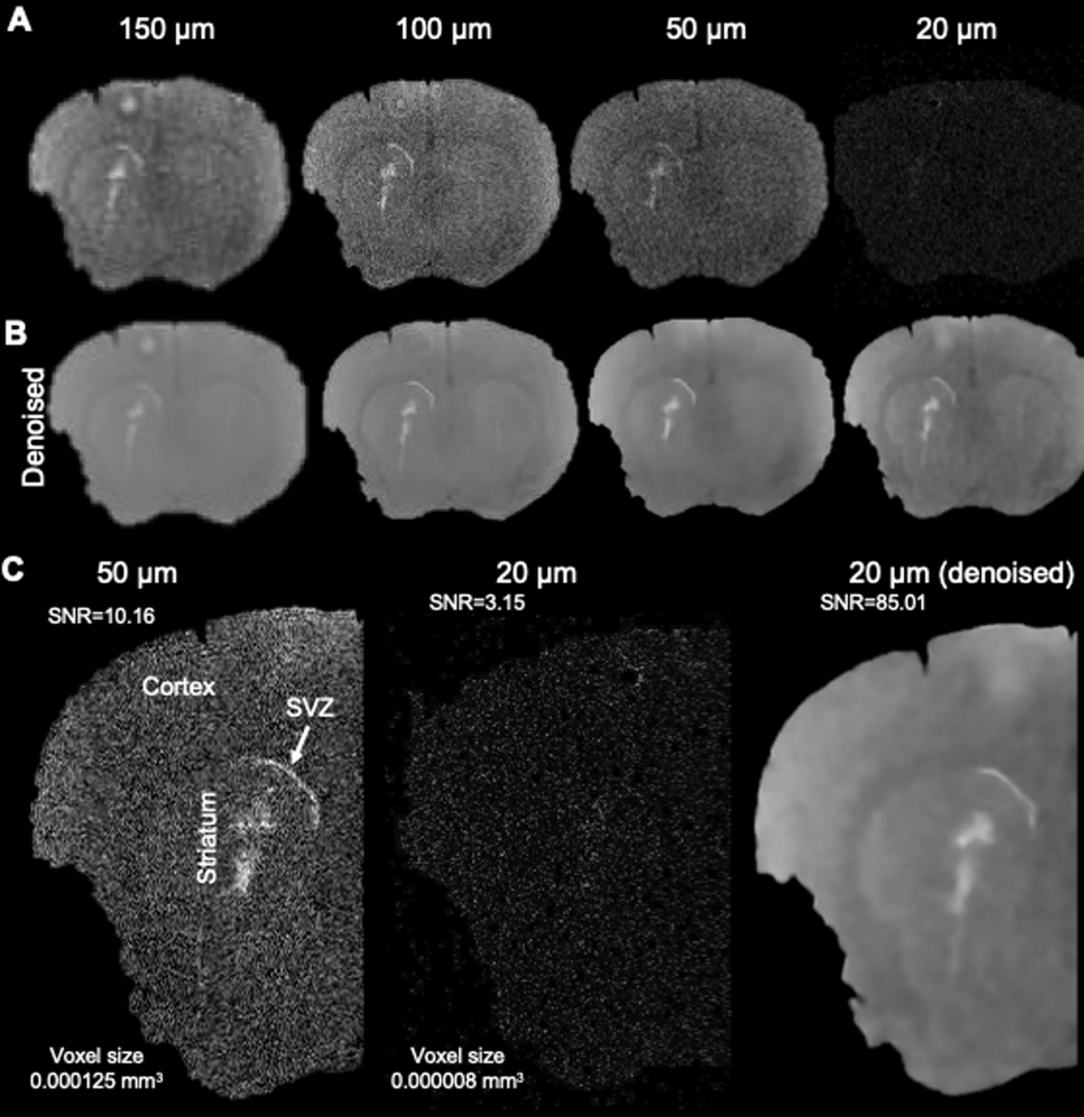
Cellular resolution MRI. (A) Injections of GdAuNP into the lateral ventricle and the striatum resulted in an increase in T_1_-weighted (T_1_w) signal on MR images. At a 150 μm isotropic resolution, the signal was readily identified and the tissue signal (*i.e.* SNR) was high. However, the T_1_w hyperintensity caused by GdAuNP was unfocussed due to partial volume effects. Increasing spatial resolution improved the localization of this hyperintensity of the GdAuNP, but SNR decreased. All scans used only 1 signal averaging. Although at 50 μm the GdAuNP hyperintensity was still visually identifiable, at the 20 μm cellular resolution tissue structure and the GdAuNP were no longer visually evident. (B) To mitigate the increase in noise at higher spatial resolution, denoising of MR images was implemented to recover the signal within these images. Denoising dramatically reduced the noise in tissue at all resolutions and provided a homogenous tissue signal. At higher resolutions (≤100 μm), especially the hyperintensity of the GdAuNP improved with a robust visualization of the lateral ventricle and its distribution in striatal tissue. The most dramatic result was evident for the 20 μm cellular resolution, for which denoising afforded a visualization of tissue structure (*e.g.* distinction between cortex, corpus callosum, striatum, ventricles), which was not feasible on the original image. Moreover, the hyperintensities induced by GdAuNP were readily distinguished from the surrounding tissues and afforded a very distinct localization. (C) Focusing on the injected hemisphere further illustrates the dramatic improvement of GdAuNP detection at cellular resolution (20 μm) using denoising to localize the signal in the lateral ventricle and the central region of the striatum.

The key challenge in cellular resolution MRM is to achieve a sufficiently high spatial resolution to be able to resolve cells within their biological context. Increasing spatial resolution provided a means to ascertain the presence of GdAuNP in both the lateral ventricle, as well as striatal tissue (Fig. 3A). T_1_w contrast was evident at a 150, 100, and 50 μm isotropic voxel resolution with a single signal average (*i.e.* NA = 1). However, the signal-to-noise ratio (SNR) decreased considerably and at a 20 μm resolution, no robust detection of GdAuNP was evident, nor was tissue contrast. To compensate for the decreasing signal, MR images were denoised to lower the background noise level. This dramatically improved the visualization of the residual tissue and GdAuNP signal. A robust T_1_w contrast between GdAuNP and brain tissue was achieved (Fig. 3B). Even at 20 μm isotropic voxel resolution, a distinct lining of the lateral ventricle was evident in the denoised image compared to both the original 50 and 20 μm resolution scans (Fig. 3C). Within striatal tissue, a clear signal contrast was evident and more robustly distinguishable from surrounding tissue, even when compared to the 50 μm resolution.

**Fig. 4:**
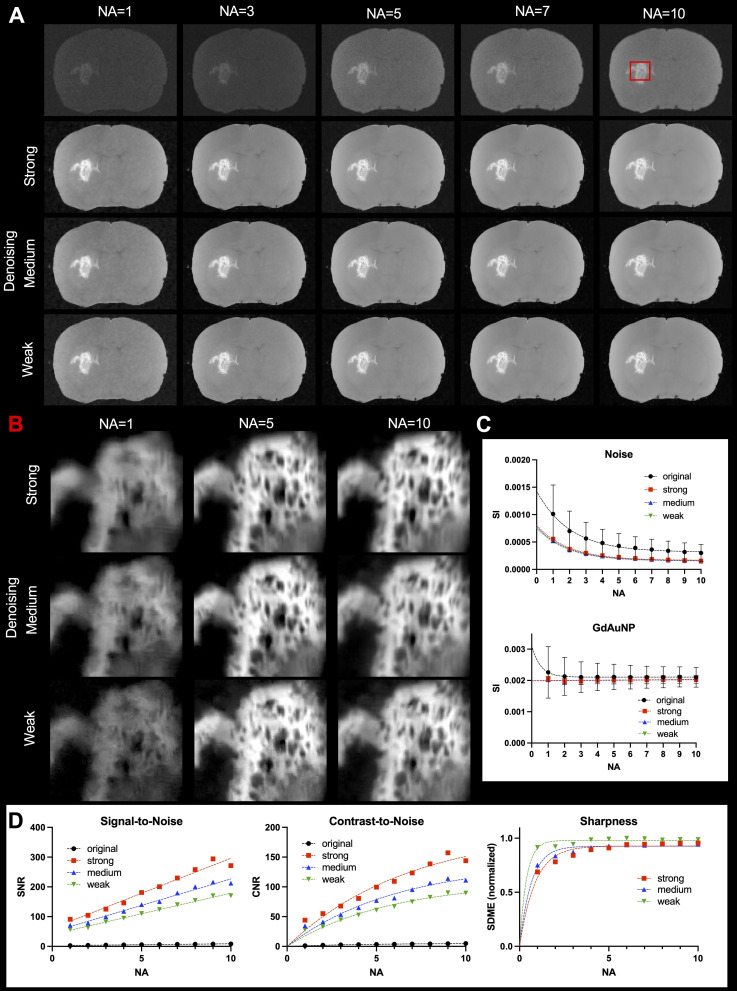
Improving signal averaging and denoising. (A) To further boost the signal at cellular resolution, signal averaging was investigated and revealed a consistent improvement in tissue signal and GdAuNP contrast. However, signal averaging comes at a substantial time penalty. Denoising significantly improved image quality even with a single average. (B) A combination of signal averaging with denoising provided the most dramatic improvements in GdAuNP detection to detail its tissue distribution. (C) Improvements in GdAuNP detection are very significantly impacted by a reduction in the noise component through signal averaging. However, a diminishing return is observed with more than 5 signal averages. Denoising overall reduces the noise component with improvements most noticeable in images with 5 signal averages. A reduction in signal variability is evident for both signal averaging and denoising, with more than 5 signal averages having diminishing returns. (D) An increase in SNR and CNR is achieved with both signal averaging and denoising. However, only minimal gains are achieved purely by using signal averaging. Denoising more significantly impacts both SNR and CNR, with an interaction of increasing signal averages evident. A strong denoising provided the most significant boost to SNR and CNR, but this came at a cost of signal sharpness (SDME). Beyond 5 signal averages, CNR revealed diminishing returns.

To further optimize the detection of GdAuNP by MRM at a ultra-high resolution (*i.e.* 20 μm isotropic voxels), signal averaging can increase signal intensities and improve detection, as well as contrast (Fig. 4A). A major improvement in signal intensity is evident with 5 signal averages. Combined with different degrees of denoising, signal averaging can achieve a high T_1_w contrast of GdAuNP with a highly specific localization within the tissue (Fig. 4B). A quantitative analysis of signal changes revealed marked reductions in the noise intensity by increasing signal averaging, but this tapered off beyond 5 signal averages (Fig. 4C). Denoising achieved a major decrease in noise measurements in line with the improvements observed with signal averaging. Strikingly, denoising also achieved a considerable reduction in variance. The same pattern of results was evident for the T_1_w signal from GdAuNP. SNR of denoised images was >20 times higher than the original images and improved linearly with more signal averages (Fig. 4D). A stronger denoising had a more marked gain in SNR. CNR was also dramatically improved with denoising, but followed a logarithmic trend that dramatically declined beyond 5 signal averages. A significant improvement in GdAuNP detection using MRM can hence be achieved using signal averaging combined with image denoising.

**Fig. 5:**
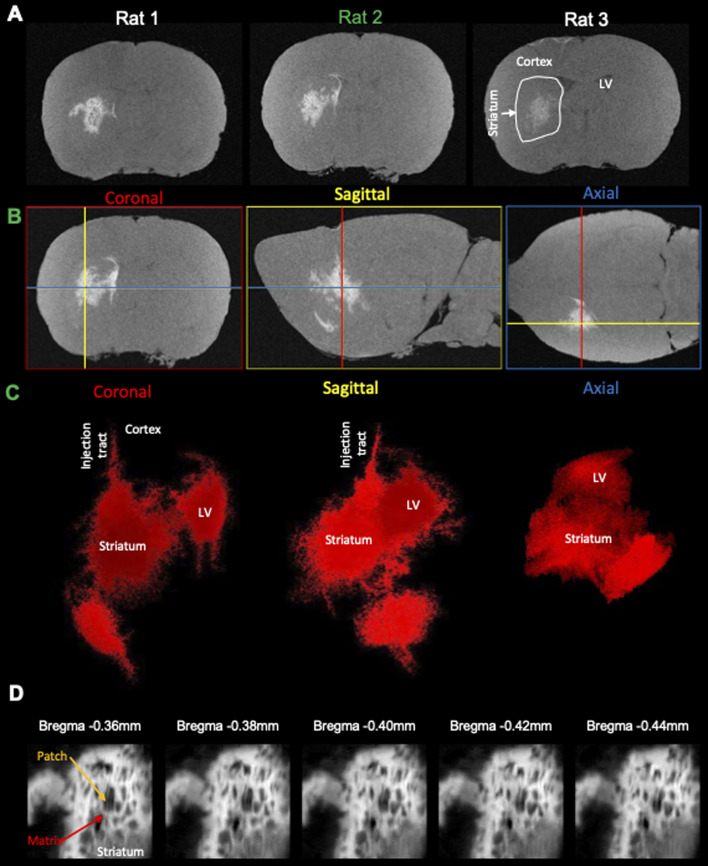
Reproducibility of isotropic cellular resolution MRI. (A) Injection of GdAuNP into the striatum and lateral ventricle of 3 rats afford the visualization of its distribution using MRI at a 20 μm isotropic resolution and image denoising. (B) The isotropic resolution affords a visualization of the GdAuNP distribution at cellular resolution in the coronal, sagittal and axial plane. An isotropic resolution is essential to accurately localize individual voxels containing GdAuNP-labeled cells in their anatomical context. (C) Otsu thresholding of these images affords the selective visualization of the GdAuNP induced tissue contrast and facilitates their localization against the background T_1_ signal of the brain. A 3D reconstruction of the GdAuNP reveals its distribution within the brain and could afford future overlays with other scans, such as T_2_ or diffusion MRI for further interrogation of specific biological questions. (D) Individual denoised and thresholded T_1_w slice images at 20 μm isotropic resolution reveal the patch-matrix of striatal tissue. Hyperintense regions correspond to the distribution of GdAuNP within the patch-matrix, whereas the region lacking T_1_ signal corresponds to the patches. Minute anatomical changes become hence discernible on the MR images. LV – lateral ventricle.

A robust cellular detection using this approach was achieved in 3 rats (Fig. 5A), affording the precise 3D localization of GdAuNP-labeled cells in the coronal, sagittal and axial plane (Fig. 5B). T_1_w thresholding afforded the separation of the brain background signal from the GdAuNP T_1_w contrast to create a 3D reconstruction of its distribution (Fig. 5C). The injection tracts are readily visible, as well as the distribution of GdAuNP labeled cells throughout the striatum. The lateral ventricle is also visible and less granular, due to GdAuNP being homogeneously distributed through the cerebrospinal fluid (CSF) after its intracerebroventricular injection. However, the region also encompasses cells in the SVZ, which are outlining the ventricular space. This approach hence provides a unique means to visualize the 3D distribution of GdAuNP-labelled cells in tissue. The 20 μm isotropic voxel resolution also visualizes the *in situ* distribution of labeled cells using T_1_w thresholding to reveal, for instance, the unique organization of the patch-matrix in the striatum (Fig. 5D). The unique pattern of T_1_w contrast distribution in this area indicates that GdAuNP are contained within the cellular compartments of the patch-matrix, rather than being homogenously distributed throughout the extracellular space.

### Verification of cellular MRM by histology

**Fig. 6:**
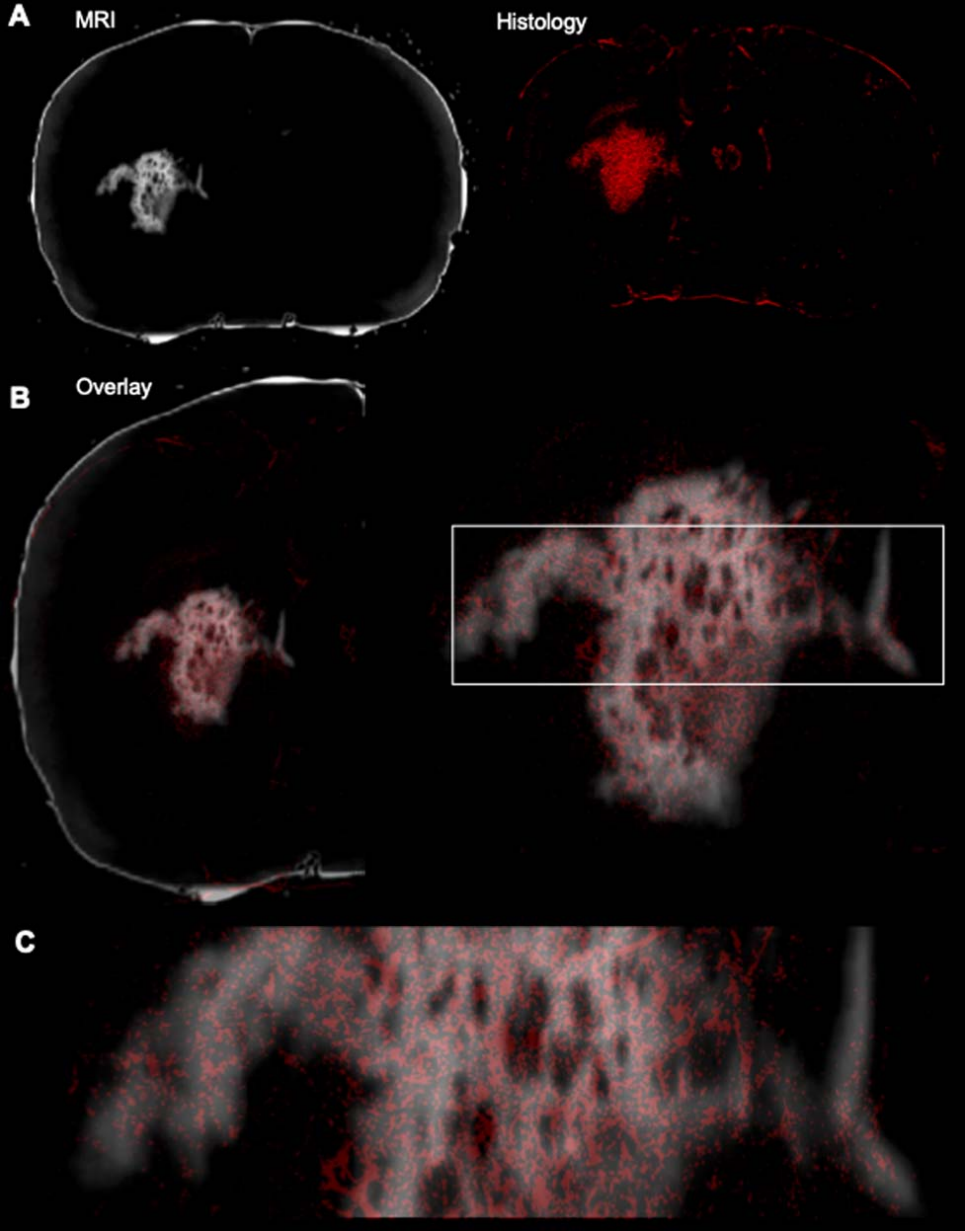
Verification of cellular resolution MRI by immunohistochemistry. (A) A macroscopic comparison of a thresholded T_1_-weighted MR image with the corresponding histological slice revealing the fluorescence moiety of the GdAuNP. (B) An overlay of both MRI and histology reveals the overlap between both imaging modalities, again confirming that T_1_ signal changes in individual voxels are caused by GdAuNP. (C) This is further highlighted in a zoomed-in region, whereas the delineation of the MRI is clearly defined by the presence of GdAuNP, hence verifying that the T_1_ increase on MR images is due to GdAuNP.

To verify that the T_1_w signal is indeed due to the presence of GdAuNP in cells, the T_1_w MRI signal was verified using immunohistochemistry. The red fluorophore conjugated on the DNA attached to the GdAuNP affords their detection using fluorescence microscopy. A macroscopic comparison between the T_1_w thresholded image and the red fluorescent whole histological slices indicates that the T_1_ signal change is reflecting the presence of GdAuNP on histological slices (Fig. 6A). An overlay further corroborated the correspondence of both imaging modalities (Fig. 6B). A closer inspection of the patch-matrix also verifies that T_1_ changes are a reflection of the differential distribution of GdAuNP in tissue (Fig. 6C). It is important to note that histological slices (50 μm) are thicker than MRI slices (20 μm).

**Fig. 7:**
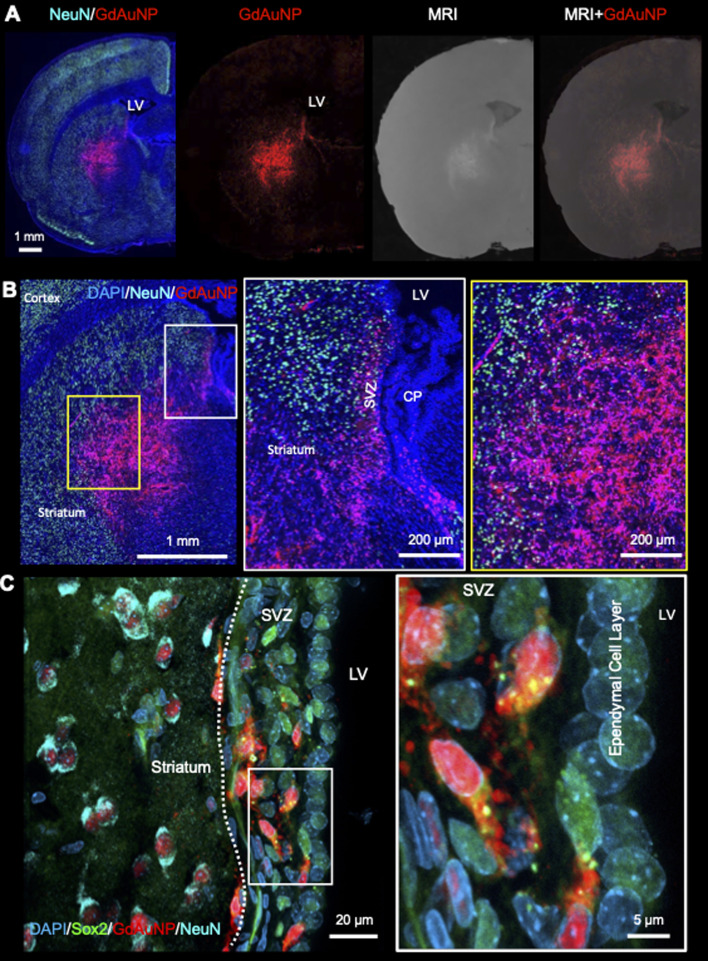
Intracellular localization of GdAuNP. (A) Macroscopic immunohistochemistry of the neuronal distribution and GdAuNP in the rat brain in comparison to the matching T_1_-weighted MR slice. An overlay of both images verifies that the MRI signal increase is due to the presence of GdAuNP. (B) GdAuNP in the striatum and the SVZ are almost completely contained within individual cells. This is further highlighted by the distribution of the GdAuNP reflecting a cellular shape rather than a homogenous signal throughout the tissue, as would be expected from the contrast material being merely distributed through the extracellular space. (C) Within the SVZ, GdAuNP were only observed within neural stem/progenitor cells, but not the ependymal cell layer, highlighting a preferential uptake into cells of the neuronal lineage. Within adjacent striatal tissue, it is also evident that GdAuNP are contained within mature neurons, which endocytosed this agent, but there is almost no agent visible within the extracellular space. LV – lateral ventricle; SVZ – subventricular zone; CP – choroid plexus.

**Fig. 8:**
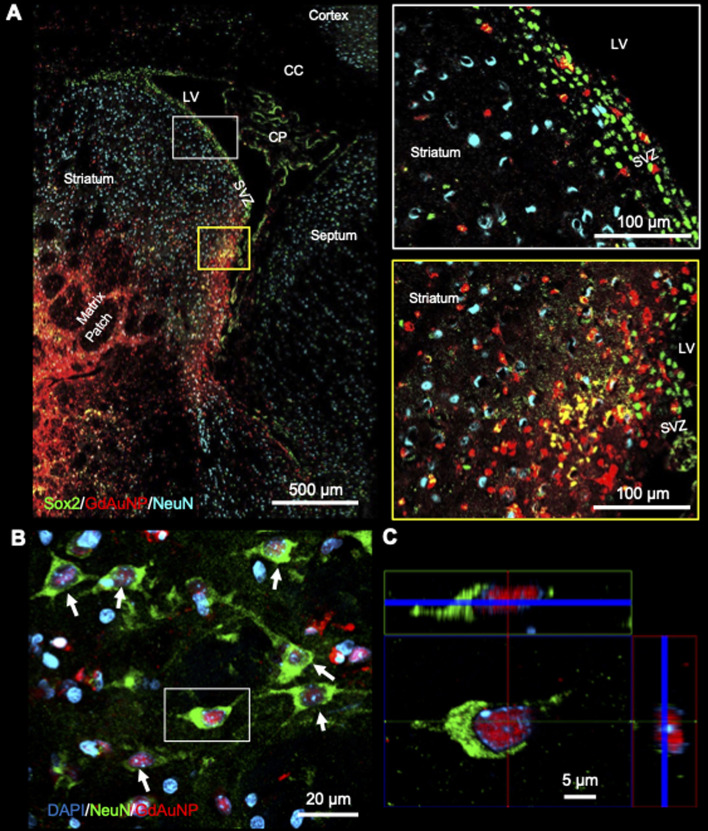
Intra-neuronal GdAuNP. (A) Neurons within the striatum and neural stem/progenitor cells within the SVZ endocytosed a high level of GdAuNP, as evidenced by the intense red intracellular fluorescence. A very negligible amount of GdAuNP remains within the extracellular space. (B) Striatal neurons readily took up the GdAuNP C. 3D reconstruction of a single neuron reveals the peri-nuclear localization of GdAuNP. LV – lateral ventricle; CC – corpus callosum; CP – choroid plexus; SVZ – subventricular zone.

**Fig. 9:**
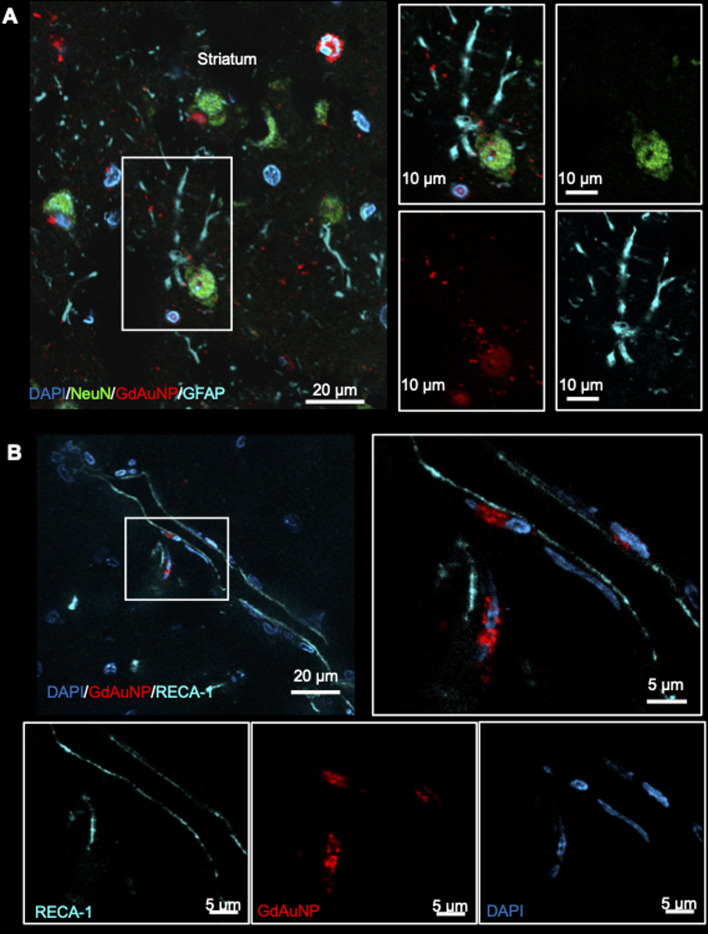
Uptake of GdAuNP in non-neuronal cell types. (A) GdAuNP associated with astrocytes were a rarity. In cases of uptake, akin to neuronal cells, the GdAuNP were found to be localized around the nucleus rather than throughout the cytoplasm or cellular extensions. (B) There was some evidence of GdAuNP along blood vessels close to the injection sites. This could be due to GdAuNP in CSF, following a glymphatic drainage route. However, in a few cases, GdAuNP could also be seen within endothelial cells.

**Fig. 10:**
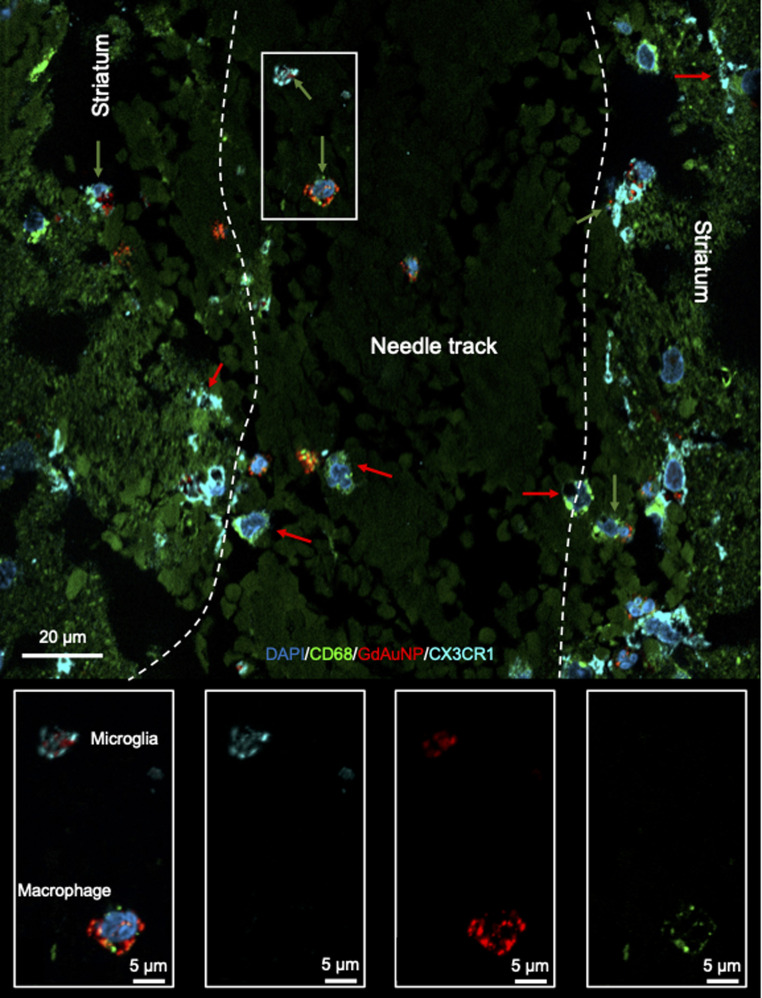
Uptake of GdAuNP in microglia and macrophages. GdAuNP were also observed in phagocytic cells within the CNS (green arrows), notably resident microglia (CX3CR1+ cells) and peripheral macrophages (CD68+ cells) that invaded in response to tissue damage caused by the injection tract. However, most macrophages and microglial do not contain GdAuNP, even at the injection site (red arrows).

Immunohistochemistry revealed individual cells, such as neurons (*i.e.* NeuN+ cells) in conjunction with GdAuNP to compare with their T_1_w localization (Fig. 7A). A cellular localization is evident in both the striatum and the SVZ (Fig. 7B). Within the SVZ, GdAuNP are observed within SOX2+ NSCs, but not ependymal cells which constitute the first layer of cells surrounding the lateral ventricle (Fig. 7C). Even within the SVZ, SOX2+ cells endocytosed GdAuNP, whereas other neighboring cells did not or only contained negligible amounts. Although macroscopically, the GdAuNP covers a large area of the striatum and SVZ, very little GdAuNP remains in the extracellular space (Fig. 8A). GdAuNP are mostly localized to the cytoplasm of cells, especially neurons, in a perinuclear location (Fig. 8B). This indicates that the T_1_w signal on MRI is due to the presence of GdAuNP inside individual cells. Although there is preferential uptake of GdAuNP into SOX2+ and NeuN+, astrocytes (Fig. 9A) and endothelial cells (Fig. 9B) also occasionally contain smaller quantities of GdAuNP. GdAuNP were also found within peripheral macrophages (CD68+) that infiltrated in response to the injection tract damage, as well as brain-resident microglia (CX3CR1+) (Fig. 10). However, within the same location there were also macrophages and microglia that did not contain GdAuNP. In all cases, almost all GdAuNP were located intracellularly, with very few GdAuNP evident in the extracellular space. These results indicate that T_1_w contrast on MR images is due to intracellular GdAuNP, predominantly in cells of the neuronal lineage.

## Discussion

Visualizing cells within tissue has remained a major challenge and requires contrast material that distinguishes cells of interest from the surrounding tissue. We here demonstrated that to detect cells at a cellular resolution using MRM, an isotropic spatial resolution of a maximum of 20 μm is required, as this is the diameter of a cells' cytoplasm, but additionally a highly efficient contrast material that is endocytosed by cells is necessary to achieve a distinction from its surrounding tissue. The design of cell-penetrating high relaxivity contrast agents, notably DNA-conjugated GdAuNP,^9,13,22^ here demonstrated an extensive *in situ* endocytosis, while affording a high level of unequivocal positive T_1_-weighted MR contrast from the surrounding tissue. Post-acquisition denoising of the MR images proved to be a valuable process to dramatically increase SNR in cellular resolution images. These multidisciplinary advances afford a paradigm shift to image individual cells in their anatomical context using MRM.

### Cellular MRI *versus* cellular resolution MRM

Although cellular MRI has been well established to visualize the distribution and migration of cells within tissue, this is typically for a population of cells at a lower spatial resolution.^3^ The detection of individual cells can be achieved using micron-sized iron oxide nanoparticles (MIONs) to create a blooming effect 50× the size of the particle and hence affords the visualization of a single labeled cell,^4,6,15,16^ but it does not afford the precise localization of that cell and the presence of a large number of cells will efface the anatomical context.

A high relaxivity contrast material is required to provide a sufficient MRI signal change that distinguishes the cell from surrounding tissue. To reduce the spatial resolution down to the size of an individual cell (*i.e.* 20 μm ^14^), a blooming effect is disadvantageous and typically T_2_-based contrast agents compete with other contrast sources (*e.g.* small bleeds, air bubbles) that can easily be mistaken for labeled cells. The design of a high relaxivity cell-penetrating T_1_ contrast agent is hence favorable to cellular resolution MRM.^13,22,23^ However, an extensive optimization of imaging parameters was required to ensure that a maximum contrast is achieved.

A physiological temperature was favorable, whereas a reduced T_1_ effect was evident close to the Curie point, as previously reported for other gadolinium based materials.^24–26^ However, T_1_-weighted contrast between GdAuNP and brain tissue was only minimally affected by temperatures above 22 °C. Although T_1_ for both brain and GdAuNP was slightly higher at 11.7 T compared to 9.4 T, the contrast between GdAuNP and brain tissue was equivalent, highlighting the potential for these agents at high field strengths. A single average of image acquisition was sufficient to achieve a good T_1_-weighted contrast at a 50 μm isotropic resolution, but at a 20 μm resolution, the GdAuNP was difficult to discern against the brain background signal.

Nevertheless, denoising of these cellular resolution images during post-processing afforded a very robust recouperation of T_1_ signal from brain tissue, as well as GdAuNP-labled cells. Increasing the number of averages for image acquisition also improved the detection of the T_1_-weighted contrast of GdAuNP, with a diminishing return beyond 5 signal averages. Denoising of these averaged images further improved detection, but as the signal in images improved, denoising exerted less dramatic effects and came at the cost of sharpness. Signal averaging is therefore advantageous over denoising for cellular resolution MRI, but denoising is extremely powerful to accommodate a limited image acquisition time.

### Verification of GdAuNP-labled cell detection using MRM

To ensure that MR images indeed reflect the presence and distribution of GdAuNP, brains were sliced to afford a visualization of GdAuNP in tissue using whole slice microscopy of the red fluorescent moiety conjugated on the GdAuNP. Fluorescence microscopy verified that the hyperintense MR signal followed the same distribution as the histological distribution of GdAuNP. To ensure that these GdAuNP were indeed inside cells, rather than in extracellular space, immunohistochemistry further visualized individual cells in brain slices and afforded a co-localization with GdAuNP. Almost all GdAuNP found within these brain slices were contained intracellularly, confirming that our MR images indeed reflected individual cells labeled *in situ* with GdAuNP.

GdAuNP efficiently endocytosed *in situ*, mostly within cells of the neuronal lineage, including neural stem/progenitor cells in the SVZ. Smaller nanoparticles are preferable for neuronal uptake (<50 nm), whereas larger particles are taken-up by microglia.^27,28^ However, the AuNP corona also plays a major role in endocytosis. Conjugation of random DNA strands to GdAuNP improves endocytosis and contributes to its perinuclear localization. The perinuclear localization, rather than lysosomal sequestration, and attachment of Gd to these DNA strands maintains GdAuNP's intracellular relaxivity,^9^ whereas endocytosis of other Gd contrast materials results in significant T_1_ quenching.^29–31^ DNA-GdAuNP previously afforded the *in vivo* imaging of transplanted NSCs using T_1_-weighted MRI^9^ and demonstrated the utility of these agents for cellular MRI. Achieving a cellular resolution using MR microscopy (MRM) can potentially usher in a new era for *in vivo* cellular and molecular imaging.

### Potential and limits of cellular resolution MR microscopy

Since the initial development of MR imaging, increasing signal and resolution has been one of the main technological challenges.^32^ However, as spatial resolution is increased the MR signal is decreased following a cubic root function. The main focus of MRI has therefore been on tissue rather than cell imaging. However, as demonstrated here, the low signal at cellular resolution can be overcome by post-processing to denoise images and improve the signal recovery.^33^ Additional improvements in acquisition can further enhance the detection of individual cells. Application of compressed sensing to the sparse signal of GdAuNP can potentially reduce image acquisition for a single average of 400 minutes to within an hour, which is feasible for *in vivo* MR microscopy in rats.^34^ To separate individual neighboring cells, an even higher spatial resolution will be required to satisfy the Abbe or Rayleigh limit of separation.^35^ An isotropic voxel at a 5 μm resolution (*i.e.* 64× higher resolution than 20 μm) would be 1/8 of a cell and sufficient to separate neighboring cells. Post-processing using super-resolution techniques, such as convex optimization,^36^ could improve cell separation and the spatial localization of individual cells within a population of labeled cells. Using extensive averaging and specialized micro-coils at high field, a 3 μm isotropic resolution is commonly considered the limit of MR microscopy due to signal detection sensitivity, translational diffusion and thermal noise.^37,38^ However, recent progress in MR imaging using dynamic nuclear polarization (DNP) at 5K indicates that at least for *ex vivo* imaging of brain samples, isotropic spatial resolutions of <2 μm might become feasible.^39^ There is an ongoing effort to bridge the gap between current microscopic MRI techniques and nanoscale tools, such as MR force microscopy, to achieve nanoscale MRI (nanoMRI) that will further improve spatial resolution and signal measurements for contrast material.^40^

Achieving a cellular resolution MRM will be especially impactful for cellular and molecular MRI, which aims to visualize how individual cells and their gene expression affect their biological context *in situ* rather than in thin histological slices. Applications that can be envisaged range from cellular positioning of cells during brain development,^41^ tracing the response of neural progenitors to brain damage,^42^ monitoring the migration of implanted NSCs,^9^ the infiltration of cells into brain tumors,^43^ to gene expression by individual cells in response to a disease stimulus.^44^ As demonstrated here, the combination of a contrast agent that has a high intracellular relaxivity and exhibits efficient *in vivo* endocytosis with a cellular resolution will offer new opportunities to investigate how individual cells contribute to tissue development and repair. MRI is a unique versatile tool that can herewith report on cellular dynamics in the intact brain that currently no other imaging technique can provide.

## Methods

### DNA–gadolinium–gold nanoparticle (DNA–Gd@Au) synthesis

**Scheme 1:**
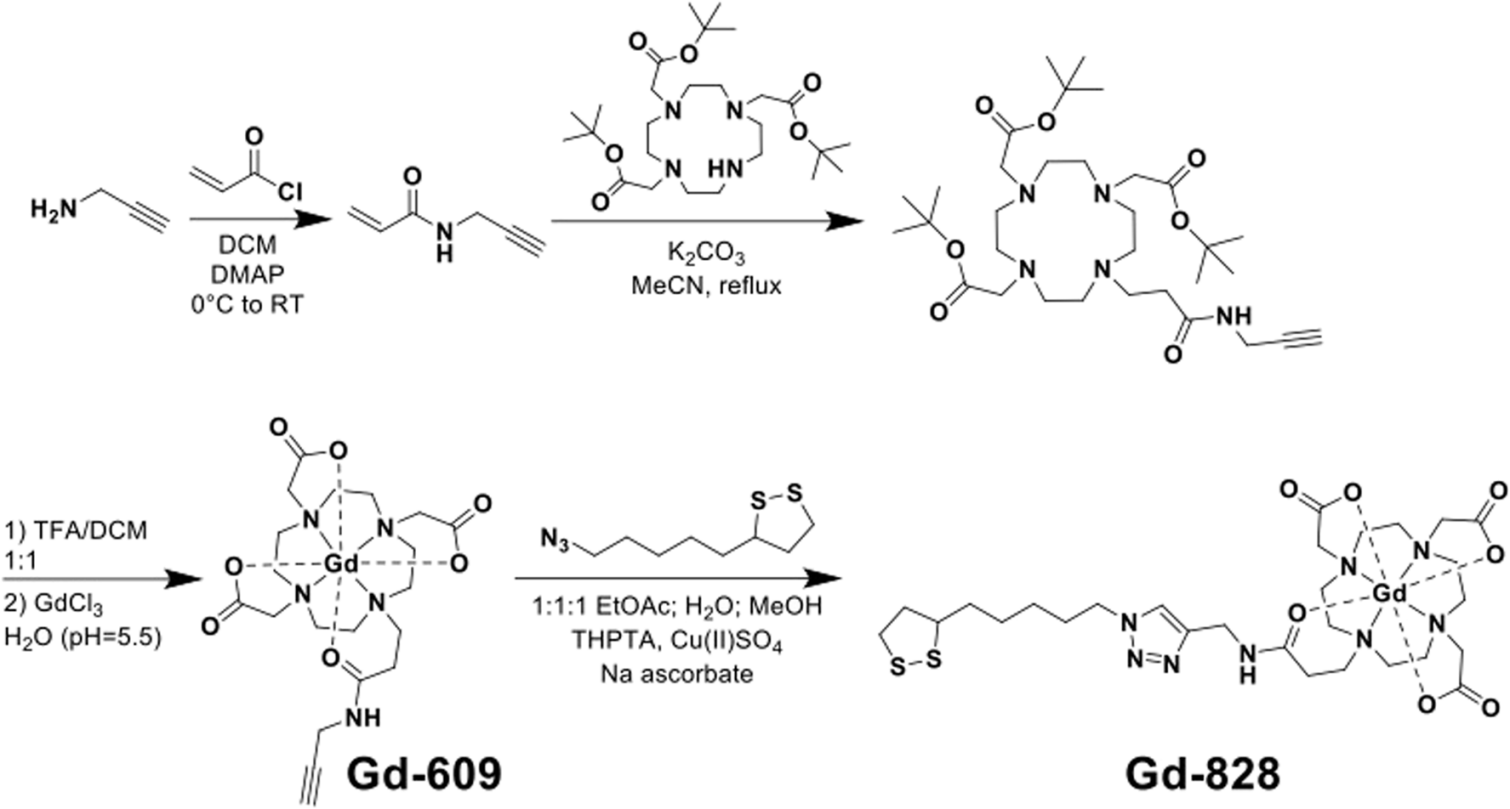
Synthesis of small-molecule Gd(iii) agents (Gd-609 and Gd-828).

**Scheme 2:**
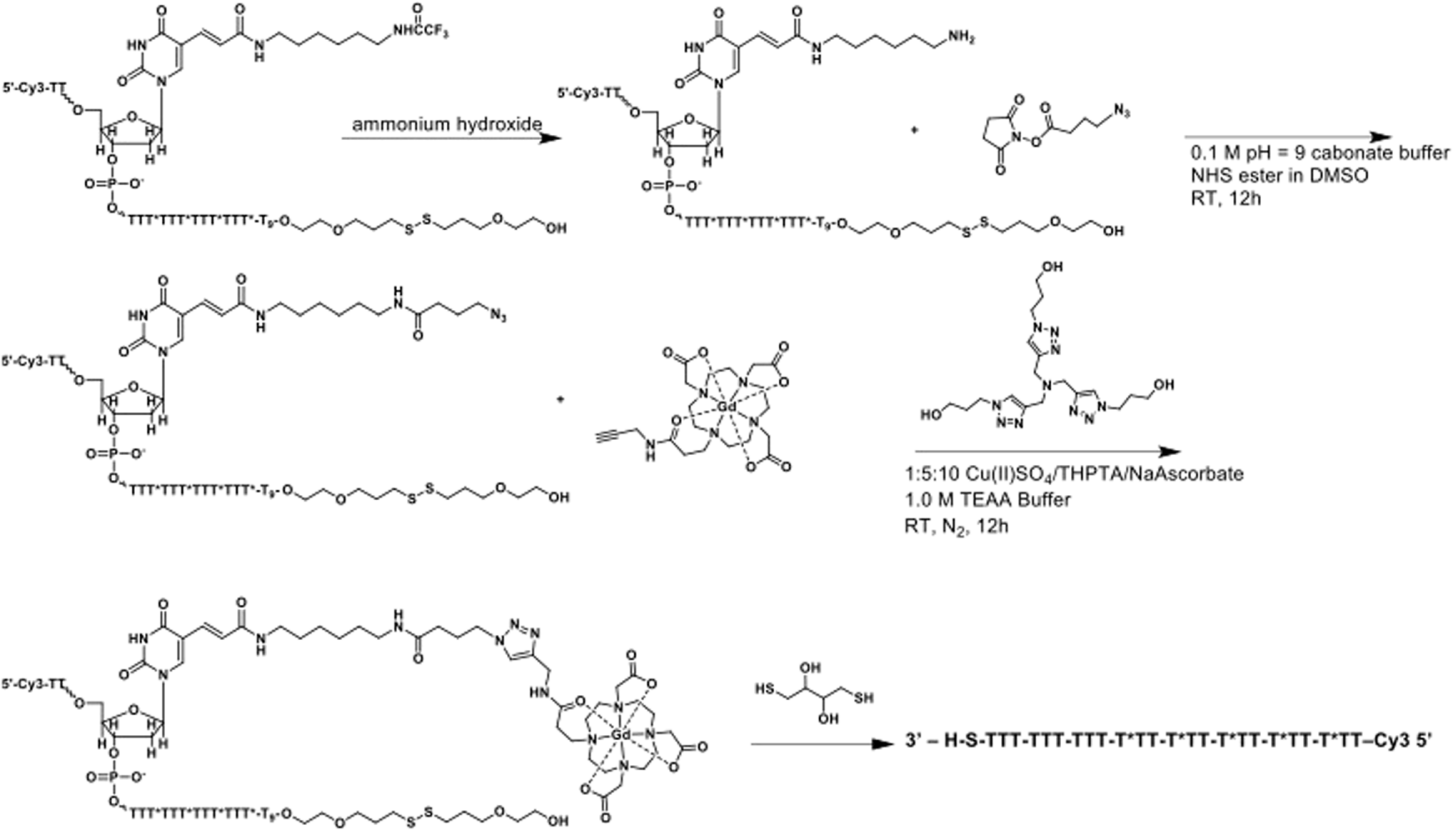
Synthesis of single stranded DNA (ssDNA) agents.

Citrate-stabilized gold nanoparticles (AuNPs) with a core diameter of 13 ± 1.4 nm were synthesized by reduction of HAuCl_4_ in refluxing water using trisodium citrate using a previously published procedure.^13,22^ Gd(III)-labeled, single-stranded DNA was synthesized using standard controlled pore glass (CPG) solid-phase support protocols with a MerMade 12 oligonucleotide synthesizer (Bio Automation) under dry Ar. All synthesizing reagents and protected 3′-Thiol modifier CPGs were purchased from Glen Research. The synthesized sequence was 3′ S-S-TTT-TTT-TTT-T*TT-T*TT-T*TT-T*TT-T*TT–Cy3 5′ where T* indicates C6 amino modifier dT modified bases and a cyanine dye (Cy3) was added as the final base using a Cy3 phosphonamidite coupled under standard conditions. Oligonucleotides were deprotected from the solid phase using AMA conditions (1 : 1 methylamine/saturated ammonium hydroxide) for 1.5 hours, followed by azide modification with azidobutyrate NHS-ester in DMSO for 12 hours at room temperature. The inorganic Gd(iii) complexes Gd-609 and Gd-828 were synthesized using previously reported procedures and HPLC purified using X-bridge C18 column^9,22^ (Scheme 1). Conjugation of the Gd-609 to the azide modified DNA was performed using Cu(i)-catalyzed 1,3 dipolar cycloaddition (click) chemistry. Each step mentioned before is purified by desalting with Sephadex G25 column (NAP 5, GE Life Sciences) and characterized by MALDI with a matrix of 2,5-dihydroxyacetophenone (DHAP) and ammonium citrate in methanol. The final DNA was HPLC purified using DNAPac™ PA200 Analytical column (4 × 250 nm) employing a gradient elution comprising tris buffer and linear gradient of salt over 17 minutes. Quantity of DNA was determined by UV-vis Cary 60 at OD 260. DNA conjugation to nanoparticles occurred after 3′ thiol deprotection with dithiothreitol (DTT) and desalting (Scheme 2). 100 equivalents of DNA were conjugated to citrate AuNPs with salt aging to 600 mM salt using five aliquots of 5 M NaCl over six hours with end-over-end rotation following a previously reported procedure,^9,22^ at which time the particles were left to rotate overnight. With 1× purification at 20 000 × *g* for 30 min, the AuNPs surface was subsequently backfilled with excess Gd-828 for 24 h. Functionalized particles were purified by spin filtration (30 kDa MWCO, Amicon® Ultra 15) in three cycles of 1500 × *g*, 10 min each. Functionalized particles were then characterized by inductively coupled plasma-mass spectroscopy (ICP-MS) for [Au] and [Gd]. The molar relaxivity (*r*_1_) of GdAuNPs was 3398 mM^−1^ s^−1^.

### Animals and DNA–Gd@Au injections

All animal procedures complied with the US Animals Welfare Act (2010) and were approved by the University of Pittsburgh Institutional Animal Care and Use Committee (IACUC). Sprague-Dawley rats (male, *n* = 3, 280 ± 15 g, Charles River Laboratories, USA) were maintained on a 12 hour light/dark schedule, with food and water available *ad libitum.* 30 μL of GdAuNP solution were injected into right lateral ventricle and 4 μL of GdAuNP solution were injected into the right striatum. Stereotaxic coordinates were determined in accordance with the rat brain atlas:^45^ anterior–posterior −0.5 mm from bregma, medial–lateral −1.5 mm from the middle line, dorsal–ventral −4.0 mm from the brain surface for the lateral ventricle; and anterior–posterior −0.5 mm from bregma, medial–lateral −3.5 mm from the middle line, dorsal–ventral −4.5 mm from the brain surface for the striatum. Nanoparticle solution was injected under isoflurane anesthesia (4% induction, 1% maintenance in 30% O_2_) using a frame-mounted injection pump (World Precision Instruments, USA) through a 50 μL Hamilton syringe with a 32 G beveled stainless-steel needle (Hamilton, USA). The injection rate was 10 μL per min for the intraventricular injection and 1 μL per min for the striatal injection; after injection the needle was staying in the brain for 10 min and then was slowly removed to minimize a chance of a liquid backflow. Lidocaine topical cream (4%, Generic) was applied as an analgesic, as well as buprenorphine (0.05 mg kg^−1^ i.p., every 12 hours) to provide sustained pain relief. After 24 hours post-implantations rats were perfusion-fixed under terminal anesthesia (1 mL kg^−1^ Fatal Plus). Blood was first flushed transcardially using ×1 PBS, then tissue was fixed by transcardial perfusion with 4% paraformaldehyde.

### MR imaging

#### MRI hardware

MRI was performed using a 9.4T/30 cm horizontal bore Bruker AV3HD MR scanner equipped with a BGA-12S HP gradient set capable of 660 mT m^−1^ maximum gradient strength and a 40 mm quadrature resonator, as well as on an 11.7 T/89 mm Bruker AV3 HD microimaging scanner with a 16-channel shim insert, a Micro 2.5 gradient insert, capable of up to 1500 mT m^−1^ maximum gradient strength, a 20 mm diameter quadrature birdcage RF coil and ParaVision 6.0.1 (Bruker Biospin, USA).

#### Optimizing TR and FA

To optimize the contrast between brain tissue and GdAuNP, regions of interest (ROIs, Supplementary Figure 1) were drawn and the T_1_ signal was measured on an *ex vivo* brain (*n* = 1) that was implanted with GdAuNP-labeled NSCs.^9^ Data for a T_1_ map was acquired on the 9.4 T MRI scanner (Fast Spin Echo with inversion recovery, TR = 10.4 s, TE-40 ms, 1 average, Flip Angle 90°, FOV 30 × 30 mm, 256 × 256 matrix, 0.117 mm in plane resolution, 35 slices at 0.5 mm slice thickness) at ambient temperature (22 °C), as well as physiological temperature (38 °C) to determine if a signal contrast occurs between *in vivo* and *ex vivo* acquisition. Temperature was controlled with a warm air system and temperature feedback next to the samples. The T_1_ maps were computed by a pixel-wise fit in Matlab. The T_1_ for ROIs containing GdAuNP labeled cells was 1241 ms at 20 °C and 1300 ms at 37 °C 1635 ms and 1741 ms for brain tissue at the same temperatures, respectively. To optimize parameters for maximum T_1_-weighted image contrast, values were used to simulate signal intensities for brain with and without GdAuNP using the equation for a steady-state signal using a spoiled gradient echo eqn (1):1
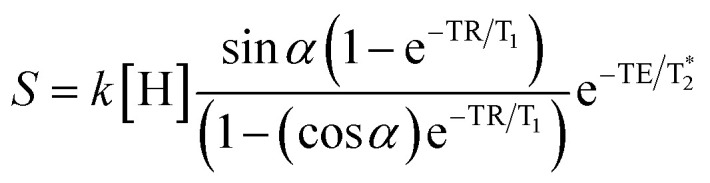


For simplicity, we assume a constant spin density *k*(*H*), and that the 
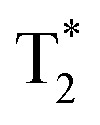
 effects 
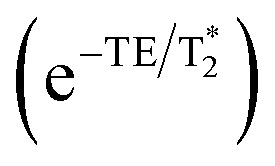
 are negligible for short TE experiments. Signal intensities were simulated for flip angles (*α*) up to 90° and TR values of 50, 100, 500 and 1000 ms. Contour maps were computed to illustrate how both TR and FA affected contrast. Verification of calculated optimal TR and FA parameters was achieved by scanning *ex vivo* brains injected with GdAuNP into the lateral ventricle and striatum at 9.4 T. A FLASH sequence (TR = 500 or 1000 ms, TE = 2.65 ms, NA = 6, Flip Angles of 10°, 30°, 50°, 70°, 90°, FOV 30 × 30 mm, 256 × 256 matrix, 0.117 mm in plane resolution, 35 slices at 0.5 mm thickness, 9 min 36 s or 19 min 20 s) was used to acquire T_1_w MR images. Mean intensities for ROIs in brain and GdAuNP areas, as well as T_1_w contrast were plotted for FA for TR = 500 ms and TR = 1000 ms.

#### Temperature and magnetic field experiments

The magnetic properties of gadolinium are affected by temperature,^24,25^ with a loss of ferromagnetism at the Curie temperature (*T*_c_) known to be approximately 292 K (19 °C). To systematically determine if a higher sample temperature improves GdAuNP contrast, samples were acquired at temperatures of 14, 18, 20, 22, 26, 30, 34, and 38 °C at 11.7 T MRI using a gradient-echo sequence (TR = 500, TE = 5 ms, NA = 8, Flip Angle 70°, FOV 23.3 × 23.3 mm, 128 × 128 matrix, 41 slices at 0.5 mm thickness, 6 min 24 s). Variable temperature control was achieved with a Bruker SmartCooler BCU-II 40/50 air chiller and a probe heater with a thermocouple feed-back loop to maintain the sample temperature to within ±0.1 °C. A comparison of signal intensity at 20 °C and 38 °C on both the 9.4 T and 11.7 T further afforded a comparison of how field strength affected the signal contrast of GdAuNPs.

#### Multi-scale resolution experiment

Although a decreasing voxel size reduces the number of ^1^H spins/voxels leading to a lower signal-to-noise ratio (SNR), the local GdAuNP concentration increases. We have previously demonstrated that smaller voxels sizes decrease SNR but yield an increased contrast from GdAuNP.^9^ An array of experiments with isotropic voxel sizes (150, 100, 50, 20 μm) was acquired at 11.7 T to determine the limits of detection (LOD) of GdAuNP by keeping the FOV constant and increasing the matrix size (Table 1 for MRI parameters). An isotropic voxel size of 20 μm was considered to achieve a cellular resolution, as we previously determined the diameter of a NSC to be 19.29 ± 0.75 μm.^14^ SNR and contrast-to-noise (CNR) were measured to determine the impact of spatial resolution on GdAuNP detection. SNR was calculated by mean tissue intensity divided by the standard deviation of noise, whereas CNR was calculated by the difference between contrast intensity and tissue intensity divided by the standard deviation of noise.

**Table 1:** Multi-scale MRI parameters (flash 3D sequence: TR = 50 ms, TE = 3.52 ms, FA = 30, FOV 12 × 24 × 16 mm, NA = 1, bandwidth 100 kHz) at 11.7 T. Signal-to-noise (SNR) was calculated by dividing the mean signal intensity by the standard deviation of the noise. Contrast-to-noise (CNR) was defined as the mean signal of the GdAuNP ROI minus the mean signal of tissue prior to division by the standard deviation of the signal in tissue

Resolution (μm)	Volume (mm^3^)	Matrix	Voxels	Time (min)	SNR (original/denoised)	CNR (original/denoised)
150	0.003375	80 × 119 × 108	1 028 160	7	47.83/161.66	14.52/147.11
100	0.001000	120 × 178 × 160	3 417 600	16	29.25/126.06	6.26/60.99
50	0.000125	240 × 356 × 320	27 340 800	64	10.16/147.61	1.53/53.30
20	0.000008	600 × 889 × 800	426 720 000	400	3.15/85.01	0.36/12.81

#### Denoising of MR images

As SNR decreases with an increased spatial resolution, post-processing aimed at denoising the image can increase the signal from an individual voxel^33^ and potentially improve the separation of GdAuNP from its background (*i.e.* brain tissue). The noise in the MRI magnitude image follows a Rician distribution^46^ with a noise variance that is nonuniform^47^ and depends on the noise-free signal intensity (signal-dependent). Therefore, the denoising framework based on the variance-stabilization transform (VST) was used^46^ and comprises three steps: (1) apply the VST to stabilize the noise variance, transforming the noise into signal-independent with an approximately Gaussian distribution; (2) denoise the image in the VST domain; (3) apply the inverse VST to take data back to its original and unbiased intensity range. The block-matching and 4D filtering (BM4D)^48^ algorithm was used in step 2 (Table 2). The BM4D is an algorithm designed for filtering Gaussian signal-independent noise of volumetric (3D) data. When denoising high-spatial-resolution data, the BM4D is very advantageous, since it is based on similarity of cubes of voxels within a search neighborhood.^49,50^ The higher the spatial resolution, the higher the similarity between cubes on the search neighborhood. Therefore, this denoising method is a great fit for our data.

**Table 2:** Parameters used for BM4D denoising algorithm

Parameter	Description	NA = 1 (Strong)	NA = 5 (Medium)	NA = 10 (Weak)
N1	Cube size (N1 × N1 × N1)	8	6	5
N2	Maximum number of similar blocks	64	64	32
Ns	Search neighborhood size (Ns × Ns × Ns)	11	11	11
lambda_thr4D	Threshold value	4.5	3.9	3.2
Thresholding	Hard or soft thresholding	‘Soft’	‘Soft’	‘Soft’
do_wiener	Flag to perform (1) or not (0) the wiener filtering step	0	0	0

#### Improving GdAuNP detection by signal averaging and denoising

To further improve the detection of GdAuNP-labeled cells at a cellular resolution (*i.e.* 20 μm isotropic), signal averaging can be employed, especially when combined with denoising methods. To determine the impact of signal averaging, 10 individual 20 μm MRI scans were acquired (*n* = 3 rat brains). Raw *k*-space data were averaged using matlab and then transformed using ParaVision to produce 10 images with NA = 1 to 10. Regions of interest for brain and GdAuNP were shared between all images to assess signal intensity, noise, as well as SNR and CNR. Due to the very low SNR of the images, the parameters of the BM4D denoising algorithm were modified from its standard version. They were optimized for NA = 1, NA = 5 and NA = 10 (respectively named as Strong, Medium and Weak) to provide a quantitative comparison for parameter selection, resulting in three denoised datasets (for each brain), one for each parameter optimization (NA = 1, NA = 5 and NA = 10). The main parameters are summarized in Table 2, and further specific details of their meaning can be found in ref. 48. In addition to SNR and CNR, the second derivative-like measure of enhancement (SDME)^51–53^ was computed, since it allows for a better analysis of the denoising results in terms of sharpness.

### Histological analyses

After removal from the skull, brains were post-fixed in 4% paraformaldehyde for 24 hours prior to being cryopreserved in 30% sucrose with sodium azide (Sigma) at 4 °C. Histological sections (50 μm thickness) were cut on a cryostat (Leica) directly onto microscopic slides to preserve the tissue morphology. Brain sections were washed 3 times with 0.01 M PBS, followed by 1 hour permeabilization and blocking in PBS + 0.1% Triton X-100 + 5% BSA (Sigma) at room temperature (21 °C). Sections were incubated overnight at 21 °C with primary antibodies (Table 3) diluted in PBS + 0.1% Triton X-100 + 1% BSA. After 18 hours, primary antibodies were washed off (3× PBS) and appropriate secondary AlexaFluor 488 or 647 antibodies (1 : 1000; Table 4) were applied for 1 hour at room temperature. Antibodies were removed by 3× washes with PBS before counterstaining with the nuclear marker Hoechst 33342 (1 μg mL^−1^, Thermo Scientific). After a further 3× washes, sections were coverslipped with Vectashield mounting medium for fluorescence and stored at 4 °C prior to imaging. Visualization of antibodies was performed with a fluorescence microscope (Axioimager M2, Zeiss) interfaced with a monochrome camera (Axiocam 820, Zeiss) driven by Zen Blue software (Zeiss) using a motorized stage.

**Table 3:** List of primary antibodies

Antibody (host)	Clone	Dilution	Company	Cat. Ref.
Hoechst 33342	A-T regions of DNA	1 : 10 000	Thermo Scientific	62249
SOX2 (rabbit)	SP76	1 : 1000	Abcam	Ab93689
NeuN (mouse)	1B7	1 : 1000	Abcam	Ab104224
GFAP (chicken)	Polyclonal	1 : 1000	Abcam	Ab4674
RECA-1 (mouse)	RECA-1	1 : 1000	Abcam	Ab9774
CD68 (mouse)	ED1	1 : 1000	Abcam	Ab31630
CX3CR1 (rabbit)	EPR24267-2	1 : 1000	Abcam	Ab303613

**Table 4:** List of secondary antibodies

Antibody (host)	Clone	Fluorochrome	Dilution	Company	Cat. Ref.
Anti-rabbit (donkey)	Polyclonal	488	1 : 1000	Abcam	Ab150061
Anti-mouse (donkey)	Polyclonal	647	1 : 1000	Invitrogen	A31571
Anti-goat (donkey)	Polyclonal	488	1 : 1000	Abcam	Ab150129
Anti-chicken (goat)	Polyclonal	647	1 : 1000	Invitrogen	A21449
Anti-goat (donkey)	Polyclonal	647	1 : 1000	Invitrogen	A21447

## Data availability

MRI scans are available from the authors upon reasonable request.

## Conflicts of interest

The authors have no personal financial or institutional interest in any of the drugs, materials, or devices described in this article.

## Supplementary Material

SC-OLF-D5SC01588J-s001

SC-OLF-D5SC01588J-s002
