# Supplementary figures and images for: Ultra-high resolution magnetic resonance microscopy of in situ gadolinium gold nanoparticle-labeled cells in the rat brain
Source: Chem Sci. 2025 Jun 3;16(27):12421–38. doi: 10.1039/d5sc01588j (PMC12131069; doi:10.1039/d5sc01588j)

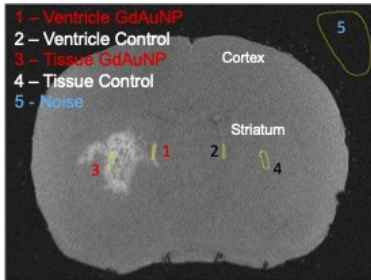

Supplement: SC-016-D5SC01588J-s001 [file SC-016-D5SC01588J-s001.pdf]
