# Supplementary material for: Ultra-high resolution magnetic resonance microscopy of in situ gadolinium gold nanoparticle-labeled cells in the rat brain
Source: Chem Sci. 2025 Jun 3;16(27):12421–38. doi: 10.1039/d5sc01588j (PMC12131069; doi:10.1039/d5sc01588j)
Supplement: SC-016-D5SC01588J-s002 [file SC-016-D5SC01588J-s002.pdf]

Supplementary Information (SI) for Chemical Science.
This journal is © The Royal Society of Chemistry 2025

Supplementary Figure Legends

Supplementary Figure 1. Regions-of-interests (ROIs) for signal-to-noise (SNR) and contrast-to-noise (CNR) measurements. To determine the impact of GdAuNP on the T1-weighted signal, ROIs were drawn to calculate the CNR between areas with and without contrast injections. To assess the SNR, a noise measurement from outside the brain was used in comparison to brain tissue.
